# Priapism, Ecstasy, and Marijuana: Is There a Connection?

**DOI:** 10.1155/2008/193694

**Published:** 2008-02-17

**Authors:** Quan T. Tran, Robyn A. Wallace, Esther H. A. Sim

**Affiliations:** ^1^School of Medicine, University of Queensland, Brisbane, Queensland 4006, Australia; ^2^Department of Internal Medicine, Princess Alexandra Hospital, Brisbane, Queensland 4102, Australia

## Abstract

Priapism is a urological emergency with multiple aetiologies including drug induced. Currently, there have been no reports of priapism induced by the combination of ecstasy and marijuana. We speculated on the potential mechanisms for acute drug-induced priapism resulting from ingestion of these two common illicit drugs.

## 1. INTRODUCTION

Priapism is a urological emergency that, left untreated, may lead to permanent damage to the corpora cavernosa resulting in erectile dysfunction. It is defined as a persistent penile erection greater than four hours in duration, which is unrelated to sexual stimulation or desire [1]. This condition can be divided into nonischaemic priapism (high-flow) and ischaemic (low-flow) priapism. Nonischaemic priapism is rare and often painless; it is thought to occur secondary to the rupture of a cavernous artery resulting in unregulated flow into the lacunar spaces and commonly results as a consequence of penile trauma or blunt perineal injury [2]. Ischaemic priapism is usually due to unremitting corporeal veno-occlusion resulting in venous stasis within the cavernous tissue and is associated with pain and tenderness. Prolonged penile veno-occlusion can result in a comprised ability to achieve an erection and penile fibrosis [2].

We present a case of a previously well 52-year-old Caucasian male who presented to the department of emergency medicine (DEM) with a four-day history of persistent erection following a night involving a sexual encounter and ingestion of ecstasy and marijuana. The next morning after his sexual encounter, he noticed a prolonged erection, which was progressing in pain. He had taken only one portion of ecstasy and marijuana but was unable to quantify the dose. He was not a regular user and had not used ecstasy or marijuana for at least 12 months. He denied taking other oral, intravenous, or intracavernous drugs. Other known causes of priapism were excluded. Two days after his sexual encounter, he presented to his family physician for treatment. Initial management with oral pseudoephedrine (60 mg) did not resolve his priapism and a needle aspiration was performed 60 minutes later. However, these measures provided only minor relief and he presented to DEM two days later.

Physical examination at DEM revealed an erect, swollen, and tender penis. No other abnormalities were detected. Detumescence was achieved by the attending urologist via insertion of 14-gauge cannulae in each side of the cavernosa. No intracavernous medications were required. The patient was discharged and referred to the medical unit to investigate any medical causes of priapism. After four weeks, there were no additional episodes of priapism. However, the patient had reported unsatisfactory erections since the incident, which may have been a consequence of his four-day delayed presentation.

## 2. DISCUSSION

We conducted a PubMed search with priapism and ecstasy (3,4-methlyenedioxymethamphetamine (MDMA)) and/or marijuana (cannabinoid, cannabis) and their pseudonyms. Only one case report was found describing a chronic ecstasy user who developed priapism following one month cessation of ecstasy use [3]. The present case differs from the latter and suggests that acute priapism is possible from a single ingestion. No specific association between priapism and marijuana was found. Therefore, we aimed to explore the acute relationship between priapism and ecstasy, marijuana, and the possibility of synergistic activities between these two substances.

Dublin and Razack [3] hypothesised that ecstasy-induced priapism is due to **the effect of ecstasy on serotonin (5-HT). Chronic use of ecstasy results in reduced serotonin activities and serotonin-related components of brain neurons [4]. In general, serotonin pathways are considered to have an inhibitory effect on sexual function [5], and chronic use of ecstasy may predispose males to priapism as a consequence of the reduced sexual inhibition [3].

However, ecstasy ingestion often results in the release of 5-HT stores causing an acute rise in 5-HT concentration [5]. Although 5-HT is primarily inhibitory, stimulation of a subset of 5-HT receptors (i.e., 5-HT_1C_, 5-HT_1D_, 5-HT_2C_) increases erections in animals [6]. Reports of priapism induced by antidepressants such as trazodone [7], citalopram [8], venlafaxine [9], and sertraline [10] are documented. The proerection properties of 5-HT_2C_ agonists are thought to be parasympathetically mediated due to 5-HT_2C_ receptors on the visceromotor neurons of the sacral parasympathetic nucleus of the spinal cord and on the dorsal gray commissure and ventral horn motor neuron [11]. Therefore, acute elevation of 5-HT induced by ecstasy ingestion may facilitate erections.

Ecstasy ingestion also results in acute dopamine (DA) release in the brain. This is thought to occur by diffusion of ecstasy into cerebral neurons stimulating DA release [5]. Dopamine has facilitative effects on sexual motivation, copulatory proficiency, and genital reflexes [11]. Clinically, levadopa (a DA precursor) was found to increase nocturnal erections in elderly males [12]. In addition, apomorphine, a dopamine (D_1_, D_2_) receptor agonist, has been observed to produce erections in men that were not accompanied by sexual arousal [13]. It is thought that the proerection properties of dopaminergic neurons occur by way of activating oxytocinergic neurons in the paraventricular nuclei and that the release of oxytocin produces erections [14].

Psychotropic drugs have been proposed as a causative agent for priapism [1]. To date, no specific case of priapism and marijuana was found in the literature. However, we have speculated on potential cannabinoid properties that may facilitate erections. Cannabinoids can potentially modulate autonomic blood outflow in both the central and peripheral nervous systems, and also have direct effects on the vasculature. The most commonly researched cannabinoid receptor is CB_1_ which is mostly found in the central nervous system but is also present in the peripheral nervous system. In rats, administered cannabinoids can cause hypotension and bradycardia, which is thought to occur via inhibition of CB_1_ mediated prejunctional sympathetic outflow (inhibition of norepinephrine), and increased vagal tone [15]. Increased parasympathetic activity would promote penile tumescence in the sexually aroused male. Furthermore, cerebral vasorelaxation has been noted as a common effect of CB_1_, CB_2_ and Δ^9^-tetrahydrocannabinol, the psychoactive ingredient of marijuana. The vasorelaxation response has been suggested to occur by way of hyperpolarization or repolarization via inhibition of Ca^2+^ mobilization in vascular smooth muscle [15]. However, the existence of cannabinoid receptors within the human penile vasculature remains unknown.

Animal studies have produced contradictory findings. Rat studies have demonstrated that cannabinoid receptor antagonists have centrally mediated proerection properties [16]. There is a paucity of studies investigating human erections and marijuana, and as a result there is insufficient evidence to suggest that marijuana will cause priapism in humans.

Synergist interactions between marijuana and ecstasy have been observed suggesting that the endocannaboid system is involved with the reinforcing properties of ecstasy [17]. This relationship may be related to the involvement of the cannabinoid in the interaction and regulation of DA synthesis and turnover [18], which is considered the principle neurotransmitter in the reinforcement pathway. Therefore, it may be possible that the DA pathway is potentiated with a combination of ecstasy and marijuana, which may also facilitate penile erections via DA stimulation of oxytocin.

## 3. CONCLUSION

We concluded that our patient suffered from an ecstasy-induced priapism, mostly likely mediated via acutely elevated dopamine and serotonin levels, and that the concentration and interaction of these neurotransmitters may have been augmented by marijuana. Cessation of these substances has resulted in no further incidences of priapism. Therefore, illicit drug use is an important consideration for acute presentations of priapism of unknown aetiology.
